# The Efficacy of Functional and Traditional Exercise on the Body Composition and Determinants of Physical Fitness of Older Women: A Randomized Crossover Trial

**DOI:** 10.1155/2019/5315376

**Published:** 2019-11-21

**Authors:** Antônio Gomes de Resende-Neto, José Carlos Aragão-Santos, Bruna Caroline Oliveira-Andrade, Alan Bruno Silva Vasconcelos, Clodoaldo Antônio De Sá, Felipe José Aidar, Josimari Melo DeSantana, Eduardo Lusa Cadore, Marzo Edir Da Silva-Grigoletto

**Affiliations:** ^1^Department of Physical Education, Center of Biological and Health Sciences, Federal University of Sergipe, São Cristóvão, Brazil; ^2^Department of Physical Therapy, Center of Biological and Health Sciences, Federal University of Sergipe, São Cristóvão, Brazil; ^3^Health Sciences Area, Unochapecó University, Chapecó, SC, Brazil; ^4^School of Physical Education, Physiotherapy and Dance, Federal University of Rio Grande do Sul, Porto Alegre, Brazil

## Abstract

**Aim:**

To analyze the efficacy of functional training (FT) and traditional training (TT) in body composition and determinants of physical fitness in older women.

**Methods:**

This is a randomized clinical trial in which participants performed two 12-week periods of different training methods, separated by eight weeks of washout. Forty-eight physically active older women (≥60 years of age) completed the intervention in three groups: (i) program that started with FT and ended with TT (FT ⟶ TT: *n* = 19), (ii) program that started with TT and ended with FT (TT ⟶ FT: *n* = 13), and (iii) stretching group (SG: *n* = 16). Before and after the interventions, the body composition was evaluated by bioimpedance, the physical fitness by battery of the Senior Fitness Test, and the quality of movement by Functional Movement Screen®.

**Results:**

Compared with SG, TT ⟶ FT and FT ⟶ TT promoted significant improvements in balance/agility (13.60 and 13.06%, respectively) and upper limb strength (24.91 and 16.18%). Only FT showed a statistically significant improvement in the strength of the lower limbs, cardiorespiratory capacity, and movement patterns when compared with SG considering the adaptations of methods separately.

**Conclusion:**

The programs used are equally effective in increasing physical fitness for daily activities in physically active older women, and therefore, they may be complementary to combat some of the deleterious effects of senescence.

## 1. Introduction

Ageing comprises a set of physiological, biochemical, and morphological changes resulting in a gradual inability for the individual to adapt to the environment [1]. This process leads to a decrease in neuromuscular function and a change in muscle contractile properties, resulting in decreased functional capacity with consequent physical dependence, frailty, and sarcopenia, often associated with increased falls, infectious processes, and other associated complications [2].

Currently, surgical and pharmacological interventions have been fundamental in several situations in the treatment and prevention of these natural declines, which could be avoided by involving older adults in regular programs of physical exercises, whose benefits are well known and have been the focus of recent research [3, 4]. When analyzing the efficacy of different exercise protocols in a systematic review, 70% of the included studies showed a reduction in the incidence of falls, 54% had improved gait ability, 80% reported increased balance, and 70% reported increased muscle strength in the frail elderly [5].

Although machine-based traditional training (TT) protocols promote several structural adaptations such as increased muscle mass [6], bone mineral density [7], and reduction of adipose tissue [8], there are questions about their ability to transfer to activities of daily living [9]. Serra-Rexach et al. [10], reported increase of strength in nonagenarian older adults after eight weeks of TT but no change was observed in the functional standing up and walking test. Moreover, recent studies show that the benefits of exercise are dependent on tasks performed during training, requiring specific movements for daily tasks to achieve greater gains in functional capacity, preventing the onset of physical disabilities [11].

In this context, functional training (FT) arises to stimulate the psychobiological system in an integral way. This method proposes the application of a systematized program of multiarticular and multiplanar exercises aimed at improving movement ability, central body strength, and neuromuscular efficiency for each individual's specific needs [12].

However, the benefits of this method are not well known in the elderly population. It is also observed the absence of a systematic training model in the studies available in the literature, as well as the lack of investigations comparing and integrating FT with TT methods, hindering a bigger analysis between the protocols used and the answers found. Thus, this experiment sought to compare the efficacy of functional and traditional training on body composition and determinants of physical fitness in physically active older women. Our initial hypothesis was that specific training protocols for daily activities carried out at an early stage are more effective in adaptive responses related to the functionality.

## 2. Methods

### 2.1. Study Design and Participants

The intervention was disseminated through leaflets, social networks, and websites of the local university, recruiting participants that met the following criteria: age between 60 and 80 years, female, practicing some type of regular physical exercise in the period of six months preceding the study, who could present the medical release term and be physically independent. Among the older women eligible for the research, the following exclusion criteria were adopted: hypertension ≥ stage 2 (systolic ≥ 160 mmHg and diastolic ≥ 100 mmHg) and musculoskeletal disorders that could restrict the practice of high-intensity exercises, these criteria, evaluated by a specialized medical team.

**Figure 1:**
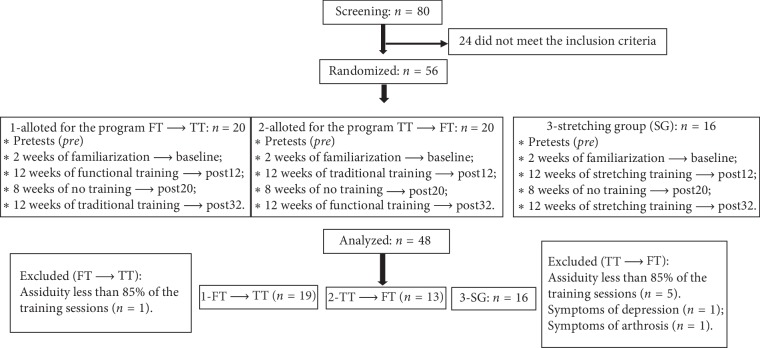
Schematic representation for screening, recruitment, allocation, and intervention.

Forty-eight older women physically active were allocated by set randomization, in which the participants were equally distributed according to their strength of lower limbs in two training programs and group that served as control: (i) program that started with FT and ended with TT (FT ⟶ TT: *n* = 19; 64 ± 4.3 years); (ii) program that started with TT and ended with FT (TT ⟶ FT: *n* = 13, 65.9 ± 5.8); and (iii) stretching group (SG: *n* = 16; 64.1 ± 3.6). Thus, participants completed two 12-week intervention periods with alternation of methods (functional/traditional) after eight weeks of no training (Figure 1).

### 2.2. Data Collection Procedures

The initial evaluation included an anamnesis with questions regarding sociodemographic aspects, health characterization, type and quantity of medication used, presence of diseases, and level of physical activity. Then, a medical evaluation was carried out to further detail the physiological parameters of each participant. They were informed about the objectives of the study, possible discomforts of the procedures, voluntary nature, right of secrecy, and possibility of withdrawal at any stage of the research, and after the acceptance of the study, they signed a free and informed consent form. Finally, the participants were advised to maintain normal dietary intake throughout the study. This study was carried out in accordance with the Declaration of Helsinki and approved by the Research Ethics Committee of the Federal University of Sergipe (No. 2.897.793/CAAE: 97652918.7.0000.5546) and also by the Brazilian Registry of Clinical Trials (RBR-9Y8KJQ).

The evaluators were blinded to the program (FT ⟶ TT or TT ⟶ FT) performed by the participants. The battery of tests was performed in five different moments and organized in the following order: body composition measurements, Functional Movement Screen, and Senior Fitness Test battery. For all performance tests, the participants were verbally encouraged to give their best.

#### 2.2.1. Anthropometry and Body Composition

Body weight was determined by a scale (Lider®, P150C, São Paulo, Brazil), with a maximum capacity of 150 kg. Height (cm) was determined using a stadiometer (Sanny®, ES2030, São Paulo, Brazil). The estimated percentage of fat, muscle mass, and basal metabolic rate were determined by electrical bioimpedance (model BC-418MA, Tanita Corporation, Tokyo, Japan). To ensure the accuracy of this evaluation, the instructions provided by the manufacturer were followed.

#### 2.2.2. Movement Patterns

The Functional Movement Screen® (FMS) was applied for analysis of movement patterns. This is a battery test involving seven functional movements that assess body mobility and stability. Each pattern was executed three times and assigned a score of 0 to 3 (1: did not perform the movement; 2: performed the movement with compensations; and 3: perfect execution). For the analyses, the total score reached by the participant was used [13, 14].

#### 2.2.3. Determinants of Physical Fitness

The Senior Fitness Test battery proposed by Rikli and Jones [15] was used to verify functional fitness, with tests that evaluate physical fitness components (flexibility, agility/dynamic balance, muscular strength of lower and upper limbs, and cardiorespiratory capacity) to perform normal daily activities in a safe and independent way, without undue fatigue.

### 2.3. Intervention

#### 2.3.1. Brief Intervention Programs

After the initial evaluations, program participants (FT ⟶ TT and TT ⟶ FT) went through two weeks of familiarization with the methods and completed 36 sessions per intervention period, lasting 50 minutes and respecting the 48-hour recovery time between training sessions. The OMNI-GSE scale was used to control and normalize training intensity between groups [16].

Stretching group: performed two sets of 20 seconds per stretch exercise for the main body parts (neck, shoulders, back, thorax, arms, wrists, hands, lower torso, hips, knees, thighs, feet, and calves) with amplitude levels articulate submaximes and relaxation practices without physical effort, with the same frequency and duration of the experimental programs.

Separately, participants of the FT performed specific exercises for their daily needs, with each session divided into four sets: (1) five minutes of mobility for the main joints (ankle, hip, and glenohumeral) and exercises for general warm up that included ten repetitions of squats and jumps; (2) 15 min of intermittent activities, organized in circuit that mainly required agility and coordination (OMNI-GSE: 6-7); (3) 25 min of multiarticular exercises for lower and upper limbs, and intense recruitment of spinal stabilizing muscles, also organized in a circuit (OMNI-GSE: 7-8); and (4) five minutes of intermittent activities (OMNI-GSE: 8-9).

In TT, the participants performed traditional exercises predominantly analytical in machines (Physicus, PLP®, Auriflama, São Paulo, Brazil), each session also divided into four sets: (1) five minutes of mobility and exercises for general warm up; (2) 15 min of continuous walking that mainly required muscular and cardiorespiratory endurance (OMNI-GSE: 6-7); (3) 25 min of resisted exercises for upper and lower limbs (squatting on the smith machine, rowing machine, leg press 45°, vertical bench press, leg curl, lat pull down, leg press, and stiff—OMNI-GSE: 7-8); and (4) five minutes of intermittent activities (OMNI-GSE: 8-9).

#### 2.3.2. Detailed Intervention Programs

The mobility exercises (1^st^ set) and the intermittent activities (4^th^ set) were performed in the same space, with only the participants of the programs (TT ⟶ FT and FT ⟶ TT). The five activities applied in the 2^nd^ set of the FT followed a density of 30 seconds of work for 30 seconds of transition between the stations. The intensity was progressive through modifications in the activities according to the level of ability and comfort:

- Medicine ball launches (2 kg): from the 1^st^ to the 18^th^ session, launches were made towards the ground, and from the 19^th^ to the 36^th^ session, vertical launches were performed on the wall at maximum concentric speed.
- Displacements with cones: from the 1^st^ to the 18^th^ session, lateral movements were performed, and from the 19^th^ to the 36^th^ sessions, short sprints were performed with a change of direction.
- Jump on a 10 cm step: from the 1^st^ to the 19^th^ session, the step up and down activity was performed, and from the 19^th^ to the 36^th^ sessions, vertical jumps were performed on the step.
- Coordinated exercises in agility ladder: from the 1^st^ to the 18^th^ session, *lateral* movements were performed, and from the 19^th^ to the 36^th^ sessions, jumps were performed.
- Alternating waves (battle rope): alternating linear movements were performed with stabilization of the shoulder girdle; every 12 sessions, the length of the rope was extended.

For logistic reasons, in the 3^rd^ set strength exercises, the individuals trained in pairs, supervised by experienced instructors, whose responsibility was to maintain the established protocols and ensure an optimal execution pattern, as well as safety and motivation. For TT, the intensity in this set was progressive by adding external loads, increased from level 6 (easy) on the OMINI-GSE scale, and with the number of repetitions performed for maintenance of 8 to 10 repetitions, that is, if the participant performed more than the maximum number of pre-established repetitions (>10), an increase of 5 to 15% in the external load was performed.

For the FT protocol, the aforementioned criterion was followed for addition of external load in the possible exercises, and modifications were made in the exercises in those performed with the own body weight, according to level of ability and comfort. The training density was 30 seconds of work per 30 seconds of transition between the stations. The modifications in the eight exercises applied in the 3^rd^ set of the FT are described below:

- Kettlebell deadlift: from the 1^st^ to the 18^th^ session, the exercise was performed with an average external load of 16 kg, and from the 18^th^ to the 36^th^ session, an external load of 20 kg was used.
- TRX suspension rowing: two *lines* parallel to the displacement of the TRX suspension with a distance of 20 cm between them were demarcated. The overload was given with the greatest inclination of the body during the sessions.
- Sit-to-stand from a 40 cm bench*:* from the 1^st^ to the 18^th^ session, the exercise was performed with the body weight, and from the 19^th^ to the 36^th^, holding a mean external load of eight kg at the chest level.
- Push-ups: from the 1^st^ to the 18^th^ session, this pushing action was performed with elastic bands (Strong Tension, ProAction®, G144, São Paulo, Brazil), and from the 19^th^ to the 36^th^, the exercise was performed in a 60 cm height.
- Farmers walk: from the 1^st^ to the 18^th^ session, the exercise was performed with an average external load of 12 kg, and from the 19^th^ to the 36^th^, it was performed with 16 kg.
- Elastic band rowing: three lines parallel to the point of attachment of the elastics were demarcated, with the first line at a distance of 40 cm and a distance of 20 cm between the others. The overload occurred with the participant positioning in the lines farthest from the point of fixation, causing greater tension in the elastic.
- Hip elevation: from the 1^st^ to the 18^th^ session, the exercise was performed with the body weight, and from the 19^th^ to the 36^th^, the movements were performed unilaterally with the knees extended and suspended alternately.
- Front plank: from the 1^st^ to the 18^th^ session, the exercise was performed in a 40 cm bench, and from the 19^th^ to the 36^th^, it was performed in a 10 cm step.

In the high-intensity intermittent exercises (4^th^ set), collective activities of executable motor complexity were used, following a 10-second work density for 20 seconds of recovery between stations and intensity equivalent to 8-9 at OMNI-GSE [17]. Here, there is a description of the two activities used to achieve this stimulation:

- Interval sprint: in a space of 30 meters, the participants were divided into five groups; three of the groups formed a column behind a cone and the other two groups formed another column, at a distance of 20 meters. Working time consisted of walking this distance with maximum speed, and recovery was achieved while the other participants in the group performed the sprints. The total volume was 8 to 12 sprints per participant.
- Tug of war: the rope was divided equally in the middle, and at each end, there was a group of participants. The activity began when groups began to perform the pull action, whose maximum strength corresponded to their work. To achieve the maximum effort in the estimated time, two coaches positioned in the middle of the rope were needed to equalize the strength between the groups. The total volume was 8 efforts per participant.

The present intervention proposal was elaborated according to the concepts presented by Da Silva-Grigoletto et al. [9] and was previously tested by Aragão-Santos et al. [18].

### 2.4. Statistical Analysis

The sample size was calculated using the G *∗* Power program (Erdfelder, Faul, and Buchner, 1996; Kiel, Germany—version 3.1.9.2) on all variables of the Senior Fitness Test battery from the results obtained by Resende-Neto et al. [19], expecting an average increase of 15% in the performance of the participants. Thus, we considered a power of 0.80 for the performed analyses for the sample size of this study.

Data were tabulated and analyzed using the Statistical Package for Social Sciences (SPSS—version 22) software. Descriptive analysis was used to summarize the general characteristics of the study participants. Data homogeneity was proven from the Levene test. The reproducibility of the functional measures was evaluated from the analysis of the interclass correlation coefficient (ICC) between initial collection and retest, adopting ≥0.85 as an acceptance criterion. For the analyzed variables, ICCs were found between 0.87 and 0.96.

The bidirectional analysis of variance for repeated measures was used to verify the differences between the interventions. When an F-ratio was significant, the Bonferroni post hoc test was used to identify where the significance occurred. The comparison between experimental groups for body composition was assessed by Student's dependent *t* test. All tests were two-tailed, and the effect size (ES) was calculated according to the equation proposed by Cohen [20], as well as the classification of each result.

The minimal clinically important difference (MCID) of each measure, determined after the intervention, was compared to assess whether intragroup changes were clinically significant. The following MCID values of measures in older adults were retrieved from the literature: 2.53 repetitions for elbow flexion, 3.3 repetitions for sit-to-stand, 1 s for time up go, 27 m for six-minute walk [21].

## 3. Results

The participant's attendance was 85% (approximately 62 sessions) for TT ⟶ FT, 95% (approximately 68 sessions) for the FT ⟶ TT and SG. Before the exercise intervention, there were no statistically significant differences between the programs in any of the analyzed variables. At the end of the two periods of 12-week training (eight weeks of no training in the middle), no statistically significant differences were observed between the TT ⟶ FT, FT ⟶ TT, and SG programs in the body composition variables (Table 1).

The programs were equally efficient in increasing physical fitness and the quality of movement patterns (*p* ≤ 0.05). When compared to SG, both TT ⟶ FT and FT ⟶ TT promoted significant improvements in balance/agility and upper limb strength. When considering TT and FT separately, only FT showed a statistically significant improvement in the strength of the lower limbs, cardiorespiratory capacity, and quality of movement patterns comparing to SG, besides a higher ES in these outcomes compared with TT. However, in the posterior chain flexibility and shoulder mobility, no differences were observed between TT ⟶ FT and FT ⟶ TT compared with SG. Also, there were no statistically significant differences between the programs in any of the evaluation moments in all outcomes analyzed (Table 2).

## 4. Discussion

This research highlights the efficacy of FT and TT in improving physical fitness for activities daily, regardless of the manipulation of the order of application of interventions. However, when analyzing the methods separately, it seems that the FT can provide greater effects than TT in lower-body strength, cardiorespiratory capacity, and quality of movement patterns.

The multisystemic adaptations evidenced by the FT ⟶ TT and TT ⟶ FT programs can be justified by the combination of different physical exercises in the same training session [22]. The organization of the session in different sets followed recommendations directed to the functionality previously published by our group [23] that besides contemplating different modalities of training in a short period of time (∼1 hour) aimed at applying these modalities in a sequence that allows gradual increase of intensity and complexity, respecting the peculiarities of the senile. Thus, there was no stagnation of effects of these training methods during the intervention period.

Although consistent investigations demonstrate the efficiency of the combination of metabolic and neuromuscular stimulation for promoting structural changes such as increased muscle mass and reduced adipose tissue [24, 25], few significant changes in body composition in active older women were observed in this study, and these adaptations may have been limited due to the absence of food control or not observed due to the low sensitivity of the instrumentation. Nevertheless, regarding muscle quality, it is worth noting that the intensity applied may not have been enough to cause clinically relevant improvement, but it was satisfactory to avoid fat gain and loss of muscle mass. Cress et al. [26] confirmed positive adaptations in muscle quality from exercises with systematization similar to the TT and FT interventions, coupled with more accurate evaluation methods.

The instability and the change of direction in the exercises of the FT can stimulate proprioceptive receptors present in the body, which provide better development of synesthetic awareness and postural control and activate stabilizing muscles with more intensity, efficiently developing the agility and balance [27, 28]. However, also with exercise, we performed the maximum concentric speed in set two, TT causes important adaptations in muscle power [29], which is a component strongly associated with dynamic balance and postural oscillation [30]. Thus, the integration of these methods may be the most effective strategy in reducing the incidence of falls and greater independence in the activities of daily living in the older women.

The integrative results of this article show similar answers of the methods regarding strength, corroborated by Cadore et al. [31] who found significant increases in muscle power, maximum dynamic and isometric strength, from the combination of strength, balance and gait exercises. The benefits of strength training, especially TT in muscle strength, are clearly evidenced in the scientific community [32]. In contrast, TF seems to act by promoting an integrated action of body structures in order to promote important neuromuscular adjustments, which result in increased strength for the performance of daily activities. In this study, these adaptations even in greater effect size than TT could be explained by the neuromuscular and metabolic specificity to the *sit-to-stand* test but also due to the greater muscular activation [33] and better functional performance [34] of exercises performed with free weights when compared with exercises performed in machines.

Concerning the cardiorespiratory capacity, it seems that the metabolic characteristic of the high-intensity interval exercises (4^th^ set), together with the circuitry and intermittent nature present in sets main of the FT (2^nd^ and 3^rd^), can promote changes in the mechanisms responsible for oxygen transport and utilization, such as increased cardiac output, mitochondrial density, and activity of oxidative enzymes [35–38], which could explain the adaptations superior to TT and SG. Corroborating this finding, Whitehurst et al. [38] also observed an increase of 7.4% in cardiorespiratory endurance after 12 weeks of functional training in circuit. It is also worth mentioning that the walking performed (2^nd^ set) by the TT was low speed, not corresponding to the speed with change of direction, required by the six-minute walking test.

Considering our results, we can affirm that the improvement of the quality of movement patterns in physically active older women seems to benefit from dynamic exercises, with greater motor complexity, specific to everyday tasks. However, Pacheco et al. [13], comparing these intervention proposals in adults and elderly physically active, did not find significant differences between methods FT and TT, also evaluated by the Functional Movement Screen, maybe due to the low intensity and complexity of the exercises applied in their intervention proposals.

In this study, the programs used were equally efficient in improving flexibility, and this adaptation could be related with the mobility exercises performed in the first set of the interventions and the accomplishment of multiarticular exercises in large amplitudes [39]. In this perspective, Correia et al. [40] state that strength training promotes important increases in range of motion through mechanisms such as reduction of joint stiffness and increased muscle elasticity in the elderly women.

Another important aspect supporting the need for integration between these two training proposals is that the older women who practiced traditional machine exercises also showed a significant increase compared with the initial values in most of the tests, suggesting that multicomponent training, with similar actions to daily activities performed at maximum concentric speed, is capable of promoting the improvement in the functionality of elderly women. In other words, the greater control in the training along with the possibility of adding external load provided by the traditional devices is also translated in improving the physical function of the older adult, as evidenced in the scientific community [25].

This investigation is focused on comparing the adaptive responses to training protocols aimed at improving functional performance in older women and presented two safe, effective, easily reproducible, and practical application methods. Despite providing important information, we recommend future studies to apply longer interventions, with a 6-month transition period between the different methods for effective washout of adaptations. We also suggest analyzing the levels of habitual physical activity and the standard food ingestion for better isolation of these intervening factors. In addition, some limitations should be taken into account when interpreting the present results: First, the small sample size and the losses to follow-up. However, losses were minimal and not expected to change the overall results. Second, the participants in our study were physically able to participate in a physical training program of high volume and intensity and thus may not be fully representative of the general older adult population.

## 5. Conclusion

Strength training programs were equally effective in improving the determinants of physical fitness and movement patterns in physically active older women, and therefore, they may be complementary to combat some of the deleterious effects of senescence. However, it appears that FT is a better option for starting health promotion programs because it provides faster adaptations and in some cases greater magnitude than TT. It is important to point out that the resistance training intervention used in the present study was designed to stimulate the different systems that promote health benefits in older people. We focused on improving the components of physical fitness and specific exercises for the daily activities, as well as providing the adequate dose of exercise against the possibilities of response to the stimulus and guarantee of optimal adaptations, respecting criteria of safety, efficacy, and functionality.

## Figures and Tables

**Table 1:** Efficacy of functional training (FT) and traditional training (TT) on the body composition of physically active elderly women.

	Moments	TT ⟶ FT (*n* = 13) 65.92 ± 5.88 years		FT ⟶ TT (*n* = 19) 64.84 ± 4.34 years		SG (*n* = 16) 64.19 ± 3.68 years
Body mass index (kg/m^2^)	*Baseline*	TT	29.22 ± 6.06	FT	29.57 ± 5.32	25.95 ± 4.68
	*Post_12_w*		28.89 ± 4.96		28.85 ± 5.74	26.08 ± 4.70
	*Post_20_w*	FT	28.96 ± 5.27	TT	28.82 ± 5.70	26.45 ± 4.61
	*Post_32_w*		28.74 ± 5.47		28.87 ± 5.66	26.03 ± 4.69

Fat (%)	*Baseline*	TT	38.61 ± 4.35	FT	38.43 ± 4.45	35.06 ± 5.40
	*Post_12_w*		37.92 ± 5.58		36.62 ± 4.60	35.63 ± 5.70
	*Post_20_w*	FT	37.92 ± 5.13	TT	38.15 ± 4.72	37.06 ± 5.50^A^
	*Post_32_w*		37.26 ± 5.05		37.02 ± 5.25	36.00 ± 6.11

Lean mass (kg)	*Baseline*	TT	39.88 ± 7.04	FT	40.17 ± 5.98	39.55 ± 6.40
	*Post_12_w*		41.27 ± 5.97		41.38 ± 5.99	39.48 ± 6.17
	*Post_20_w*	FT	41.12 ± 7.40	TT	40.71 ± 6.37	38.30 ± 6.52
	*Post_32_w*		41.28 ± 7.55		41.74 ± 6.30^A^	39.15 ± 6.31

Basal metabolic rate	*Baseline*	TT	1214.00 ± 224.03	FT	1254.01 ± 194.74	1203.31 ± 193.99
	*Post_12_w*		1252.07 ± 185.92		1260.36 ± 179.30	1200.37 ± 188.02
	*Post_20_w*	FT	1254.76 ± 226.83	TT	1249.21 ± 201.79	1185.75 ± 201.33
	*Post_32_w*		1255.76 ± 229.64		1282.05 ± 202.28	1181.06 ± 196.09

Values presented in mean and standard deviation (M ± SD). ^A^*p* ≤ 0.05  vs. baseline. w: weeks. SG: stretching group.

**Table 2:** Efficacy of functional training (FT) and traditional training (TT) on the physical fitness related to daily activities and the quality of movement patterns of physically active elderly women.

	Moments	TT ⟶ FT (*n* = 13)			FT ⟶ TT (*n* = 19)	SG (*n* = 16)
Sit and reach (cm)	*Baseline*	TT	2.80 ± 6.92	FT	1.76 ± 6.29	2.62 ± 9.83
	*Post_12_w*		5.84 ± 7.04^A^		4.71 ± 7.12^A^	5.21 ± 11.42^A^
	*∆%—ES*		108.57–0.44^*∗*^		167.61–0.47^*∗*^	98.85–0.26^*∗*^
	*Post_20_w*	FT	6.25 ± 5.78	TT	3.73 ± 7.84	3.50 ± 8.87
	*Post_32_w*		9.59 ± 5.21^AC^		6.36 ± 7.70^AC^	5.96 ± 9.42^C^
	*∆%—ES*		53.44–0.58^*∗∗*^		70.51–0.34^*∗*^	70.29–0.28^*∗*^

Back scratch (cm)	*Baseline*	TT	−4.36 ± 6.68	FT	−4.60 ± 6.26	−1.04 ± 6.59
	*Post_12_w*		−2.82 ± 6.29^A^		−3.48 ± 6.31^A^	0.08 ± 6.91^A^
	*∆%—ES*		54.61–0.23^*∗*^		32.18–0.18	107.69–0.17
	*Post_20_w*	FT	−3.68 ± 6.63	TT	−6.24 ± 6.82^B^	−0.73 ± 6.96
	*Post_32_w*		−1.95 ± 6.63^C^		−4.30 ± 6.70^C^	1.06 ± 7.09^C^
	*∆%—ES*		47.01–0.26^*∗*^		31.09–0.28^*∗*^	245.21–0.26^*∗*^

Time up go (sec)	*Baseline*	TT	5.11 ± 0.69	FT	5.04 ± 0.54	4.82 ± 0.53
	*Post_12_w*		4.57 ± 0.48^A+^		4.32 ± 0.36^A+^	5.10 ± 0.61
	*∆%—ES*		10.57–0.78^*∗∗*^		14.29–1.33^*∗∗∗∗*^	5.81 to −0.53
	*Post_20_w*	FT	4.59 ± 0.54^A^	TT	4.65 ± 0.39^B^	4.71 ± 0.56^B^
	*Post_32_w*		4.19 ± 0.63^ABC+^		4.21 ± 0.35^AC+^	4.76 ± 0.53^B^
	*∆%—ES*		8.71–0.74^*∗∗*^		9.46–1.13^*∗∗∗*^	1.06 to −0.09

Sit-to-stand (rep)	*Baseline*	TT	16.38 ± 3.37	FT	15.68 ± 2.45	15.95 ± 3.19
	*Post_12_w*		19.00 ± 4.22^A^		20.89 ± 3.19^A+^	16.50 ± 2.19
	*∆%—ES*		16.00–0.78^*∗∗*^		33.23–2.13^*∗∗∗∗*^	3.45–0.17
	*Post_20_w*	FT	18.23 ± 2.61	TT	18.26 ± 3.55^AB^	17.25 ± 2.64
	*Post_32_w*		21.61 ± 3.54^ABC+^		21.00 ± 4.59^AC+^	16.50 ± 2.09
	*∆%—ES*		18.41–0.95^*∗∗∗∗*^		15.01–0.77^*∗∗*^	−4.35 to −0.28

Elbow flexion (rep)	*Baseline*	TT	19.92 ± 4.28	FT	19.52 ± 3.68	18.90 ± 3.44
	*Post_12_w*		22.73 ± 4.47^A+^		23.39 ± 3.57^A+^	18.68 ± 3.73
	*∆%—ES*		14.11–0.66^*∗∗*^		19.83–1.05^*∗∗∗*^	−1.16 to −0.06
	*Post_20_w*	FT	22.46 ± 2.96^A^	TT	22.13 ± 3.36^A^	22.71 ± 3.96^AB^
	*Post_32_w*		27.07 ± 3.08^ABC+^		25.18 ± 3.46^AC+^	21.67 ± 3.52^AB^
	*∆%—ES*		20.53–1.56^*∗∗∗∗*^		13.78–0.91^*∗∗∗*^	−4.58 to −0.26

Six-minute walk (m)	*Baseline*	TT	536.93 ± 59.21	FT	551.33 ± 51.30	549.59 ± 51.11
	*Post_12_w*		563.11 ± 51.47		590.49 ± 40.27^A+^	548.46 ± 54.79
	*∆%—ES*		4.88–0.44^*∗*^		7.10–0.76^*∗∗*^	−0.21 to −0.02
	*Post_20_w*	FT	553.74 ± 53.17	TT	572.41 ± 44.87	566.73 ± 40.44
	*Post_32_w*		587.34 ± 48.62^AC+^		593.47 ± 44.08^AC+^	541.44 ± 50.66^C^
	*∆%—ES*		6.07–0.63^*∗∗*^		3.68–0.47^*∗*^	−4.46 to −0.63

Functional Movement Screen (points)	*Baseline*	TT	9.38 ± 2.29	FT	9.00 ± 2.64	8.75 ± 2.32
	*Post_12_w*		10.15 ± 2.30		12.00 ± 2.02^A**+**^	9.93 ± 2.54
	*∆%—ES*		8.21–0.34^*∗*^		33.33–1.14^*∗∗∗*^	13.49–0.51^*∗∗*^
	*Post_20_w*	FT	10.23 ± 1.87	TT	9.36 ± 3.13^B^	9.75 ± 2.95
	*Post_32_w*		11.53 ± 2.50^A^		10.78 ± 2.78^AC^	9.93 ± 3.02
	*∆%—ES*		12.71–0.70^*∗∗*^		15.17–0.45^*∗*^	1.85–0.06

Values presented in mean and standard deviation (M ± SD). Post_12: 12 weeks of functional training, post_20: 8 weeks of no training, and post_32: 12 weeks of traditional training. ^A^*p* ≤ 0.05 vs. baseline, ^B^*p* ≤ 0.05 vs. post_12, ^C^*p* ≤ 0.05 vs. post_20, ^+^*p* ≤ 0.05 vs. SG, ^≠^*p* ≤ 0.05 vs. TT, and ^#^*p* ≤ 0.05 vs. FT. SG: stretching group. w: weeks. Δ%: percent change. ES: effect size: ^*∗*^small: 0.2; ^*∗∗*^median: 0.5; ^*∗∗∗*^large: 0.8; ^*∗∗∗∗*^very large: >1.3.

## Data Availability

The data used to support the findings of this study are available from the corresponding author upon request.
